# Affinity of Antifungal Isoxazolo[3,4-*b*]pyridine-3(1*H*)-Ones to Phospholipids in Immobilized Artificial Membrane (IAM) Chromatography

**DOI:** 10.3390/molecules25204835

**Published:** 2020-10-20

**Authors:** Krzesimir Ciura, Joanna Fedorowicz, Petar Žuvela, Mario Lovrić, Hanna Kapica, Paweł Baranowski, Wiesław Sawicki, Ming Wah Wong, Jarosław Sączewski

**Affiliations:** 1 Department of Physical Chemistry, Faculty of Pharmacy, Medical University of Gdańsk, Al. Gen. J. Hallera 107, 80-416 Gdańsk, Poland; hanna.kapica@gumed.edu.pl (H.K.); pawel.b1995@gmail.com (P.B.); wsawicki@gumed.edu.pl (W.S.); 2 Department of Chemical Technology of Drugs, Faculty of Pharmacy, Medical University of Gdańsk, Al. Gen. J. Hallera 107, 80-416 Gdańsk, Poland; joanna.fedorowicz@gumed.edu.pl; 3 Department of Chemistry, National University of Singapore, 3 Science Drive 3, Singapore 117543, Singapore; petar.zuvela@nus.edu.sg (P.Ž.); chmwmw@nus.edu.sg (M.W.W.); 4 Know-Center, Inffeldgasse 13, AT-8010 Graz, Austria; mlovric@know-center.at; 5 Department of Organic Chemistry, Faculty of Pharmacy, Medical University of Gdańsk, Al. Gen. J. Hallera 107, 80-416 Gdańsk, Poland; jaroslaw.saczewski@gumed.edu.pl

**Keywords:** immobilized artificial membrane, IAM-HPLC, isoxazolo[3,4-*b*]pyridin-3(1*H*)-one, isoxazolone

## Abstract

Currently, rapid evaluation of the physicochemical parameters of drug candidates, such as lipophilicity, is in high demand owing to it enabling the approximation of the processes of absorption, distribution, metabolism, and elimination. Although the lipophilicity of drug candidates is determined using the shake flash method (*n*-octanol/water system) or reversed phase liquid chromatography (RP-LC), more biosimilar alternatives to classical lipophilicity measurement are currently available. One of the alternatives is immobilized artificial membrane (IAM) chromatography. The present study is a continuation of our research focused on physiochemical characterization of biologically active derivatives of isoxazolo[3,4-*b*]pyridine-3(1*H*)-ones. The main goal of this study was to assess the affinity of isoxazolones to phospholipids using IAM chromatography and compare it with the lipophilicity parameters established by reversed phase chromatography. Quantitative structure–retention relationship (QSRR) modeling of IAM retention using differential evolution coupled with partial least squares (DE-PLS) regression was performed. The results indicate that in the studied group of structurally related isoxazolone derivatives, discrepancies occur between the retention under IAM and RP-LC conditions. Although some correlation between these two chromatographic methods can be found, lipophilicity does not fully explain the affinities of the investigated molecules to phospholipids. QSRR analysis also shows common factors that contribute to retention under IAM and RP-LC conditions. In this context, the significant influences of WHIM and GETAWAY descriptors in all the obtained models should be highlighted.

## 1. Introduction

Isoxazolone derivatives have numerous medicinal applications, mainly due to their antibacterial [1,2,3], antitubercular [1], fungicidal [4], antileukemic [1], anticancer [5], antioxidant [6,7,8], antiandrogenic [9], and antinociceptive activities [7], among many others [10,11,12].

The studied isoxazolo[3,4-*b*]pyridine-3(1*H*)-one derivatives show moderate antibacterial potencies and noticeable antifungal activity against *Candida parapsilosis* (minimum inhibitory concentration (MIC) < 6.2 g/mL) [2,5]. The search for new antifungal agents represents a serious task in medicinal chemistry due to the development of resistance to the currently used antifungal drugs, as well as their high toxicity. Hence, treatment of fungal infections remains a major challenge for modern medicine.

Assessment of the physicochemical properties of drug candidates, such as lipophilicity, is highly desirable, since it allows the estimation of potential absorption, distribution, metabolism, and elimination properties. Consequently, effective selection of the most suitable drug candidates for further analyses is feasible. Traditionally, lipophilicity is determined using the shake flash method (*n*-octanol/water system) or reserved phase liquid chromatography (RP-LC). However, more biosimilar alternatives to classical lipophilicity measurements are available nowadays. One of the alternatives is immobilized artificial membrane (IAM) chromatography. The IAM stationary phases consist of phosphatidyl choline residues covalently bound to silica, which mimics the phospholipid membrane monolayer. IAM chromatography shows superior biomimetic properties to RP-LC since phosphatidylcholine is the major phospholipid present in cell membranes. The first HPLC columns with an immobilized artificial membrane were developed and patented by Pidgeon et al. in 1989 [13]. Currently, IAM.PC.DD2 columns have been proposed by Regis Technologies. The unprecedented advantage of this technique is that it much more accurately imitates in vivo conditions compared with the octanol–water system [14]. IAM-HPLC has been successfully applied for phospholipid affinity assessments of beta-blockers [15], calcium channel blockers [16], local anesthetics [17], biogenic amines [18], and sets of structurally non-related basic, acidic and neutral drugs [19]. This biomimetic method has also been used to evaluate more complex properties than phospholipid affinity, such as the capability to cross the blood–brain barrier [15], oral absorption [20], volume of distribution [21], and skin permeation [22].

The present study is a continuation of our research focusing on the physiochemical characterization of biologically active isoxazolo[3,4-*b*]pyridine-3(1*H*)-one derivatives [23]. The main aim of this study was to assess the affinity of isoxazolone derivatives to phospholipids using IAM-HPLC. Next, the similarities and differences between chromatographically established phospholipid affinities and lipophilicity parameters were examined. Finally, quantitative structure–retention relationship (QSRR) modeling of IAM retention was performed using differential evolution coupled with partial least squares (DE-PLS) regression. The proposed QSRR model presents the influence of the physicochemical properties of the studied isoxazolones on their retention under IAM conditions and allows estimations of the affinity to phospholipids for newly synthesized isoxazolone derivatives.

## 2. Results and Discussion

### 2.1. Analysis of IAM Chromatographic Data

Retention of analytes in IAM high-performance chromatography (IAM-HPLC) can be determined in various ways, depending on the elution method. Two of the most popular approaches to outline the IAM-HPLC retention of molecules include: extrapolated log*k*_wIAM_ parameters in the case of isocratic elution and IAM chromatographic hydrophobicity indices (CHI_IAM_) dedicated to gradient elution. Generally, CHI indices were originally proposed by Valko et al. for predicting lipophilicity using RP-LC [24]. Further studies carried out by the same group adapted the CHI method to IAM-HPLC conditions [14]. Briefly, CHI/CHI_IAM_ indices determined using a fast-organic phase gradient (mostly acetonitrile) are derived from an assumption that analytes do not move in the chromatographic system until a suitable organic phase concentration reaches the column, which starts elution of the analytes practically within the dead time [25]. Thus, the CHI/CHI_IAM_ values range from 0 to 100, corresponding to the concentration of acetonitrile in the mobile phase and reflecting the nature of the target compound: low and high values correspond to hydrophilic and lipophilic molecules, respectively. The fast gradient procedures provide significant advantages over isocratic elutions, since they avoid multiple isocratic measurements and extrapolation procedures. Consequently, the CHI/CHI_IAM_ indices can be determined within a few minutes. Another advantage of CHI/CHI_IAM_ approaches involves the standardization of data, which results from applying a set of reference compounds with known CHI/CHI_IAM_ values. For the reasons presented above, we determined the affinities to phospholipids of a library of bioactive isoxazolo[3,4-*b*]pyridine 3(1*H*)-ones derivatives, previously synthesized by our group, using IAM-HPLC and the protocol proposed by Valko et al. The chemical structures and names of the investigated compounds are presented in Table S1, whereas the determined retention times of the reference substances and the investigated analytes are listed in Tables S2 and S3, respectively. Finally, Table 1 displays the CHI_IAM_ values determined for the studied isoxazolone derivatives.

The obtained CHI_IAM_ values ranged from 7.29 to 34.30, except for Compound 14, which migrated together with the front of the mobile phase, avoiding detection under IAM conditions. Generally, the tested isoxazolone derivatives featured rather low affinity to phospholipids. Compound 17, which was the only fluorine-containing analyte among the tested molecules, showed the lowest affinity to phospholipids, with a CHI_IAM_ parameter of 7.29. In contrast, the derivatives that showed the highest phospholipid affinity included compounds with large alkyl substituents, namely the benzyl (**6**) and butyl (**13**) derivatives of pyrido-isoxazolone, with CHI_IAM_ values of 34.01 and 34.30, respectively. The 3,5-dimethoxybenzyl congener 11 also demonstrated a high CHI_IAM_ value of 33.98. Surprisingly, the significant differences between the assessed CHI_IAM_ indices for Compounds 15–17, which demonstrated substantial antifungal activities, suggest that IAM measurements may not be adequate for estimation of the antifungal potential of drug candidates within this class of compounds.

### 2.2. Quantitative Structure–Retention Relationship Modeling

**Figure 1:**
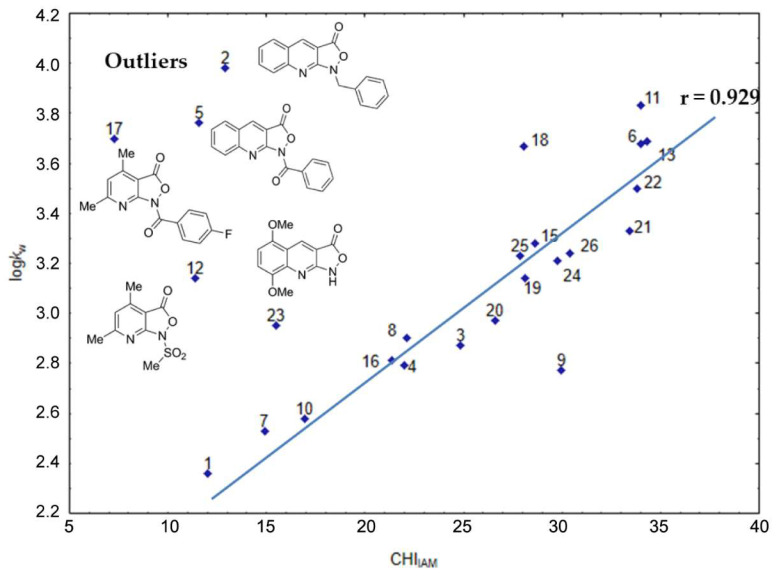
Plot of the CHI_IAM_ and log*k*_w_ chromatographic indices of the studied isoxazolone derivatives.

To recognize the retention mechanism of the studied isoxazolone derivatives in detail under IAM chromatography conditions, the QSRR approach was implemented. The QSRR approach, which was introduced to chromatographic research by Kaliszan [27], quantitatively links the molecular descriptors characterizing the molecules with their retention parameters. As several studies for structurally related compounds have reported a linear relationship between IAM retention and lipophilicity [28,29,30], we verified the correlation between the RP-LC log*k*_w_ parameters determined previously and the CHI_IAM_ indices established in this study. The plot of CHI_IAM_ and log*k*_w_ indices is presented in Figure 1.

The obtained results indicate that for some compounds within the series, the affinity for phospholipids is not properly related to lipophilicity since, based on the 2.5 sigma-rule, five outliers were identified. Although a linear trend was observed upon exclusion of the outliers (*r* = 0.929), lipophilicity does not fully explain the retention mechanism of the compounds investigated in the IAM chromatographic experiments. For the above reason, a QSRR analysis was performed. Hence, to formulate a QSRR model that would illustrate the relationships between the physicochemical properties and the retention parameters, the DE-PLS approach was implemented. First, the dataset of 2848 descriptors was reduced to 278 based on a set of strict pre-selection criteria [31]. Five outlying compounds (1, 2, 3, 15, and 19) were identified and removed from the set of data. Subsequently, the dataset was randomly split into 70% as the training set and 30% as the testing set. This DE-PLS scheme produced the final optimal model with 11 descriptors (listed in Table 2) and explained by four latent PLS variables (LVs).

**Figure 2:**
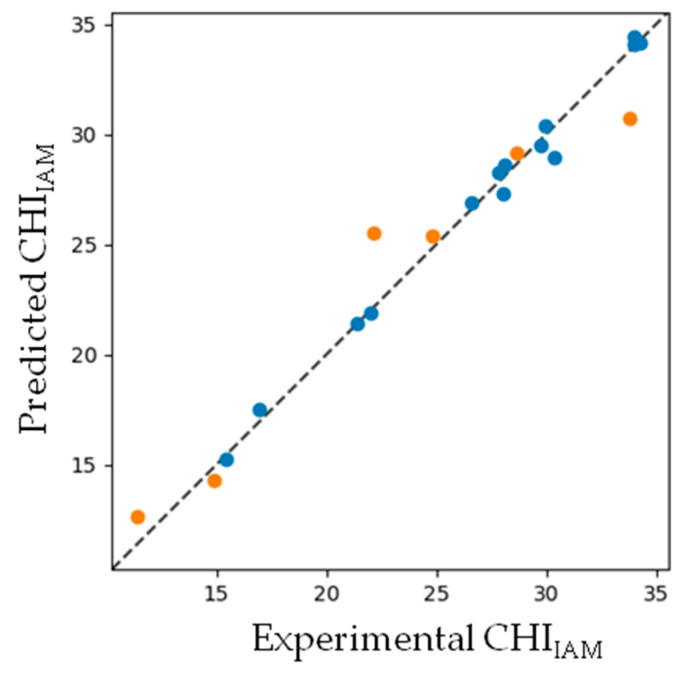
CHI_IAM_ predictive ability plot of the DE-PLS model.

The four LVs explained 66.20% of the variance in the X-space and 94.10% of the variance in the Y-space of PLS. The model yielded strong performance (Figure 2) with a root mean square error (RMSE) of 0.533 in the training set and 1.983 in the testing set. The corresponding values of the coefficient of determination (*R*^2^) were 0.992 and 0.933 for the training and testing sets, respectively.

**Figure 3:**
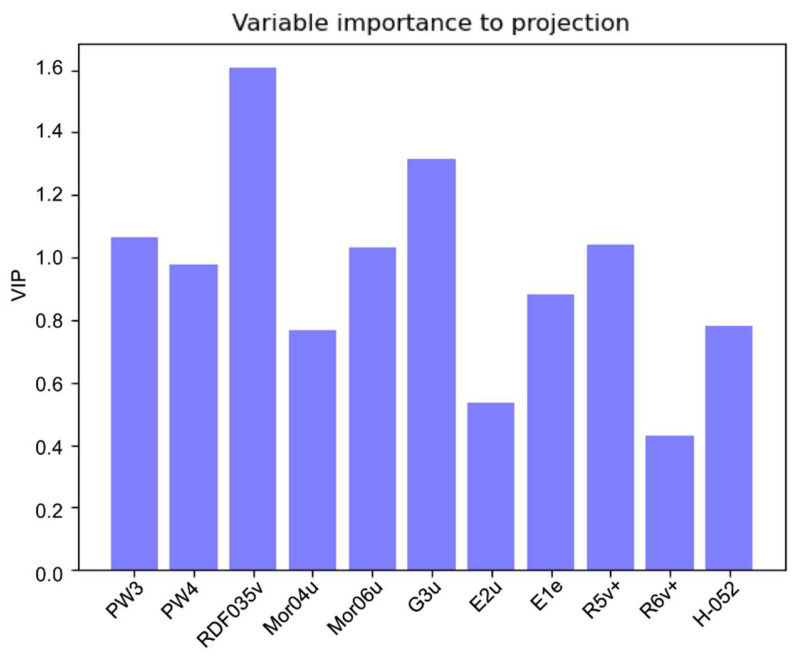
Variable importance to projection (VIP) plot for the DE-PLS model.

From Figure 3 (the PLS variable importance to projection (VIP) plot), except for Mor04u, E2u, and R6V+, most of the molecular descriptors had similar importance in predicting retention time on the IAM column.

From our investigation of the nature of the molecular descriptors, which determine the retention under IAM conditions, some general observations regarding the molecular mechanisms of the interactions between the isoxazolone derivatives and phospholipids can be drawn. The descriptors related to lipophilicity are not included in the proposed QSRR model. However, 3 of 11 valid descriptors that significantly affected the retention parameters include information attributed to van der Waals volume. The largest group of descriptors included in the obtained model are WHIM descriptors, which incorporate information regarding molecular size, shape, symmetry, and atomic distribution. GETAWAY descriptors, which are comparable with WHIM parameters [32], significantly influence the obtained QSRR model. Generally, GETAWAY descriptors encode information regarding the 3D molecular geometry and order derived from the molecular influence matrix (MIM) calculations. Notably, all model GETAWAY descriptors included in the QSRR are weightings of van der Waals volume. Through comparing the proposed DE-PLS model with the previously obtained GA-PLS models [23] describing the RP-LC retention parameters of the studied isoxazolone derivatives, some conclusions can be generalized. Similarities in the nature of the valid descriptors are evident. Hence, in RP-LC models, WHIM and GETAWAY descriptors also play an important role in QSRR analyses. In all models, the descriptors that belong to 3D-MoRSE class are present. However, a significant difference of the QSRR models pertains to the lack of polarizability-related descriptors in the IAM scheme, even though it has been shown previously that these descriptors noticeably influence the retention parameters in RP-LC models. Chemically advanced template search (CATS) descriptors, which comprise information regarding lipophilicity, are not included in the QSRR model describing the IAM chromatographic separations, whereas these descriptors have been shown to impose a significant impact on retention under RP-LC conditions [23].

## 3. Materials and Methods

### 3.1. Reagents

Dimethyl sulfoxide (DMSO) (purity: ≥99.7%) and acetonitrile (gradient grade for liquid chromatography, LiChrosolv^®^), sodium phosphate dibasic dehydrate and sodium phosphate monobasic monohydrate were purchased from Sigma-Aldrich (Steinheim, Germany). Ultrapure water, obtained with the Millipore Direct-Q 3 UV Water Purification System (Millipore Corporation, Bedford, MA, USA), was utilized for purification of the water used for preparation of the buffer mobile phase.

### 3.2. Analytes

The analytical standards of the model substances used for CHI_IAM_ determination were purchased from Alfa Aesar (Haverhill, MA, USA), Sigma-Aldrich (Steinheim, Germany), and Acros Organic (MA, United States). Detailed information regarding the reference substances’ suppliers is listed in Table S3. Synthesis and purification of the pyrido- and quinolino-isoxazolones have been described previously [2,4,26]. The structures of the investigated hybrids are presented in Table S1. All the studied compounds were dissolved in DMSO to obtain a concentration of 1 mg/mL and stored at 2–8 °C prior to analyses.

### 3.3. HPLC Analysis

All the HPLC experiments were carried out using a Prominence-1 LC-2030C 3D HPLC system (Shimadzu, Japan) equipped with a Diode-Array Detection (DAD) detector and an IAM.PC.DD2 column (10 × 4.6 mm; particle size 10.0 µm with an IAM guard column; Regis Technologies, Morton Grove, IL, USA). The LabSolution system (version 5.90, Shimadzu, Japan) was applied to control the HPLC system and for data collection. Before analysis, stock solutions of solutes were diluted to obtain concentrations of 100 μg/mL; the injected volume was 5 μL. The IAM-HPLC analyses were carried with a linear gradient of 0–85% phase B (where phase A was a 10 mM phosphoric buffer at pH 7.4 and phase B was acetonitrile) at a flow rate of 1.5 mL/min. The temperature of the chromatographic column was controlled and set to 30.0 °C and the analysis time was 6.5 min. The CHI_IAM_ indices of the studied compounds were obtained using a calibration set of reference substances using the protocol proposed by Valko et al. [33]. Each HPLC analysis was run in triplicate.

### 3.4. QSRR Analysis

The molecular descriptors were calculated for the geometries obtained previously [23]. Briefly, the structures were optimized with the Gaussian 16 package by means of density functional theory (DFT) at the PBE0/6-311G++(2df,2pd) level of theory [34] and the SMD solvation model [35]. Next, the molecular descriptors were derived using Dragon 7.0 (Talete, Milan, Italy) software. The full names, symbols, and definitions of the descriptors can be found the handbook by Todeschini et al. [36]. Subsequently, the calculations pertaining to the QSRR analysis were performed in Python (Python Software Foundation, Beaverton, Delaware, USA) using the DE-PLS algorithm.

## 4. Conclusions

The investigated isoxazolone derivatives show relatively low affinities to phospholipids. The results suggest that for the studied group of structurally related isoxazolones, significant divergences occur between the retention parameters established under IAM and RP-LC conditions. Although some correlations between CHI_IAM_ and log*k*_w_ can be found, lipophilicity does not completely explain phospholipid affinity within the studied chemical group. The QSRR analysis also indicated common factors that condition retention under IAM and RP-LC conditions, since a significant influence of WHIM and GETAWAY descriptors in all the obtained models was found. Although several studies have shown that IAM chromatography can be a useful tool for scoring various biological activities [15,21,22], unfortunately, the obtained results indicate that this approach does not allow for rapid assessment of the antifungal activity of the tested compounds.

## Figures and Tables

**Table 1:** Chromatographically determined CHI_IAM_ and log*k*_w_ parameters [23], accompanied by MIC values against *Candida* species [2,4,26].

No.	CHI_IAM_	log*k*_w_	*Candida* *albicans*	*Candida* *parapsilosis*	*Candida* *glabrata*	*Candida* *lusitaniae*	*Candida* *tropicalis*
1	12.04	2.36	>128	>128	>128	>128	>128
2	12.92	3.98	>128	>128	>128	>128	>128
3	24.85	2.87	>128	>128	>128	>128	>128
4	22.02	2.79	>128	>128	>128	>128	>128
5	11.60	3.76	>128	>128	>128	>128	>128
6	34.01	3.68	100	>200	>200	100	>200
7	14.91	2.53	>200	>200	>200	100	>200
8	22.14	2.90	>200	>200	>200	>200	>200
9	29.99	2.77	50	50	50	50	50
10	16.92	2.58	>200	>200	>200	>200	>200
11	33.98	3.83	>200	100	>200	>200	>200
12	11.40	3.14	100	>200	>200	100	>200
13	34.30	3.69	*nt*	*nt*	*nt*	*nt*	*nt*
14	*nd*	2.07	>200	>200	>200	50	100
15	28.65	3.28	100	<6.2	>200	50	25
16	21.39	2.81	100	<6.2	>200	>200	>200
17	7.29	3.70	50	<6.2	100	25	100
18	28.04	3.67	>200	100	>200	>200	>200
19	28.10	3.14	>128	>128	>128	>128	>128
20	26.61	2.97	>128	>128	>128	>128	>128
21	33.40	3.33	>128	>128	>128	>128	>128
22	33.78	3.50	>128	>128	>128	>128	>128
23	15.46	2.95	>128	>128	>128	>128	>128
24	29.78	3.21	>128	>128	>128	>128	>128
25	27.84	3.23	>128	>128	>128	>128	>128
26	30.37	3.24	>128	>128	>128	>128	>128

*nd*—not detected; *nt*—not tested.

**Table 2:** List of molecular descriptors selected by the DE-PLS model.

No.	Symbol	Full Name	Descriptor Type
1	PW3	path/walk 3—Randic shape index	Topological indices
2	PW4	path/walk 4—Randic shape index	Topological indices
3	RDF035v	Radial Distribution Function—035, weighted by van der Waals volume	RDF descriptors
4	Mor04u	signal 04, unweighted	3D-MoRSE descriptors
5	Mor06u	signal 06, unweighted	3D-MoRSE descriptors
6	G3u	3rd component symmetry directional WHIM index, unweighted	WHIM descriptors
7	E2u	2nd component accessibility directional WHIM index, unweighted	WHIM descriptors
8	E1e	1st component accessibility directional WHIM index, weighted by Sanderson electronegativity	WHIM descriptors
9	R5V+	R maximal autocorrelation of lag 5, weighted by van der Waals volume	GETAWAY descriptors
10	R6V+	R maximal autocorrelation of lag 6, weighted by van der Waals volume	GETAWAY descriptors
11	H-052	H attached to C0(sp3) with 1 × attached to next C	Atom-centered fragments

## Supplementary Materials

The following are available online. Table S1: Chemical names and structural formulas of the studied of pyrido- and quinolino-isoxazolones. Table S2: The retention data of the investigated substances. Table S3: The IAM-HPLC retention data of model substances utilized for determination of the CHIIAM values.
