# Supplementary material for: Affinity of Antifungal Isoxazolo[3,4-b]pyridine-3(1H)-Ones to Phospholipids in Immobilized Artificial Membrane (IAM) Chromatography
Source: Molecules. 2020 Oct 20;25(20):4835. doi: 10.3390/molecules25204835 (PMC7587931; doi:10.3390/molecules25204835)
Supplement: Supplementary file 1 [file molecules-25-04835-s001.pdf]

Supplementary materials

Affinity of Antifungal Isoxazolo[3,4-b]pyridine 3(1H)-ones to Phospholipids in Immobilized Artificial Membrane (IAM) Chromatography

Krzesimir Ciura[1*], Joanna Fedorowicz[2], Petar Žuvela[3], Mario Lovrić[4], Hanna Kapica[1], Paweł Baranowski[1], Wiesław Sawicki[1], Ming Wah Wong[3], Jarosław Sączewski[5]

[1] Department of Physical Chemistry, Faculty of Pharmacy, Medical University of Gdańsk, Al. Gen. J. Hallera 107, 80-416, Gdańsk, Poland
[2] Department of Chemical Technology of Drugs, Faculty of Pharmacy, Medical University of Gdańsk, Al. Gen. J. Hallera 107, 80-416, Gdańsk, Poland
[3] Department of Chemistry, National University of Singapore, 3 Science Drive 3, Singapore 117543, Singapore
[4] Know-Center, Inffeldgasse 13, AT-8010 Graz, Austria
[5] Department of Organic Chemistry, Faculty of Pharmacy, Medical University of Gdańsk, Al. Gen. J. Hallera 107, 80-416, Gdańsk, Poland

Table 1S. Chemical names and structural formulas of the studied of pyrido- and quinolino-isoxazolones

No.	Chemical name	Chemical structure
**1**	Isoxazolo[3,4-*b*]quinolin-3(1*H*)-one	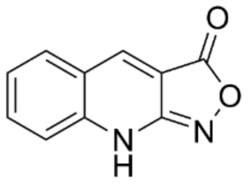
**2**	1-Benzylisoxazolo[3,4-*b*]quinolin-3(1*H*)-one	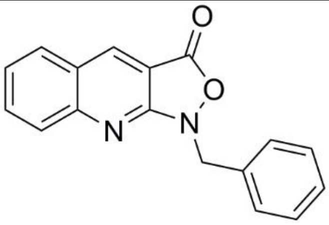
**3**	1-Methylisoxazolo[3,4-*b*]quinolin-3(1*H*)-one	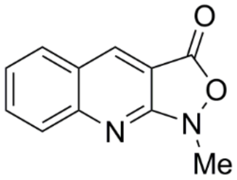
**4**	1-Acetylisoxazolo[3,4-*b*]quinolin-3(1*H*)-one	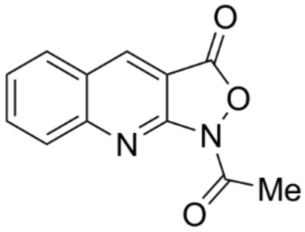
**5**	1-Benzoylisoxazolo[3,4-*b*]quinolin-3(1*H*)-one	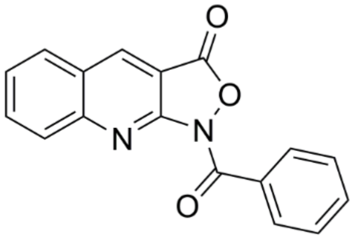
**6**	1-Benzyl-4,6-dimethylisoxazolo[3,4-*b*]pyridin-3(1*H*)-one	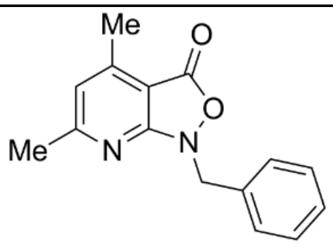

**7**	1-Acetyl-4,6-dimethylisoxazolo[3,4-*b*]pyridin-3(1*H*)-one	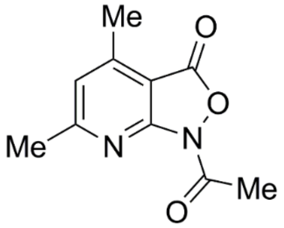
**8**	1-Ethyl-4,6-dimethylisoxazolo[3,4-*b*]pyridin-3(1*H*)-one	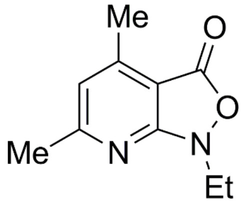
**9**	1-Benzoyl-4,6-dimethylisoxazolo[3,4-*b*]pyridin-3(1*H*)-one	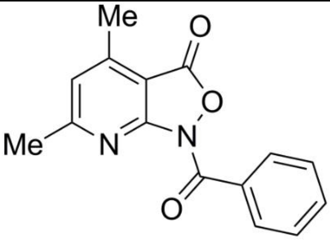
**10**	1,4,6-Trimethylisoxazolo[3,4-*b*]pyridin-3(1*H*)-one	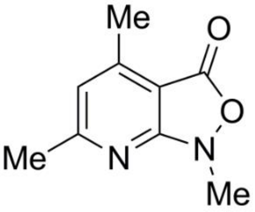
**11**	1-(3,5-Dimethoxybenzyl)-4,6-dimethylisoxazolo[3,4-*b*]pyridin-3(1*H*)-one	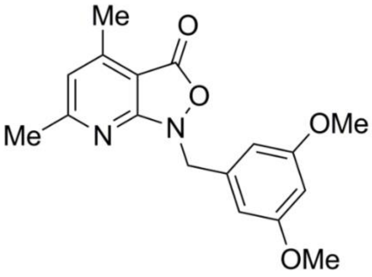
**12**	4,6-Dimethyl-1-(methylsulfonyl)isoxazolo[3,4-*b*]pyridin-3(1*H*)-one	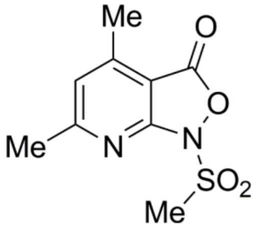

**13**	1-Butyl-4,6-dimethylisoxazolo[3,4-*b*]pyridin-3(1*H*)-one	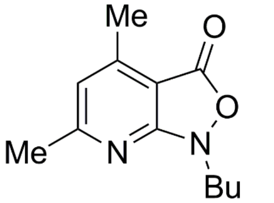
**14**	4,6-Dimethylisoxazolo[3,4-*b*]pyridin-3(1*H*)-one	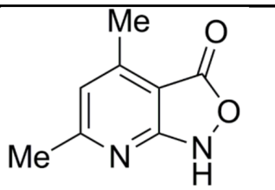
**15**	4,6-Dimethyl-1-propylisoxazolo[3,4-*b*]pyridin-3(1*H*)-one	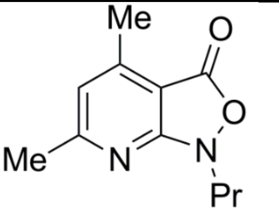
**16**	4,6-Dimethyl-1-(prop-2-yn-1-yl)isoxazolo[3,4-*b*]pyridin-3(1*H*)-one	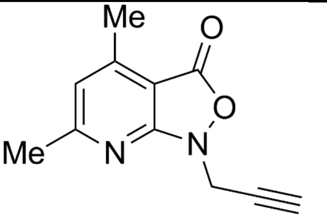
**17**	1-(4-Fluorobenzoyl)-4,6-dimethylisoxazolo[3,4-*b*]pyridin-3(1*H*)-one	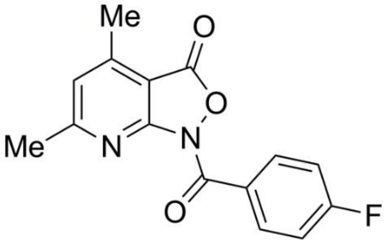
**18**	1-(3,4,5-Trimethoxybenzyl)-4,6-dimethylisoxazolo[3,4-*b*]pyridin-3(1*H*)-one	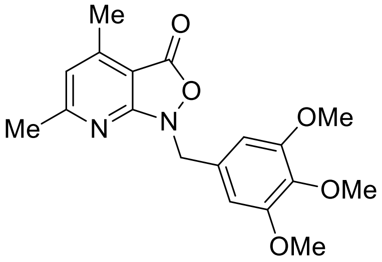
**19**	6-Methoxy-1-methylisoxazolo[3,4-*b*]quinolin-3(1*H*)-one	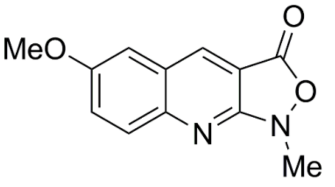

**20**	6-Fluoro-1-methylisoxazolo[3,4-*b*]quinolin-3(1*H*)-one	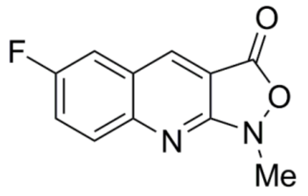
**21**	6-Chloro-1-methylisoxazolo[3,4-*b*]quinolin-3(1*H*)-one	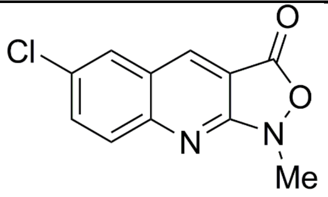
**22**	1,6,7-Trimethylisoxazolo[3,4-*b*]quinolin-3(1*H*)-one	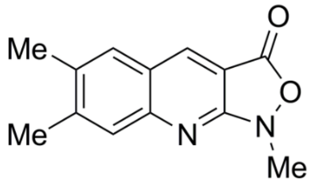
**23**	5,8-Dimethoxyisoxazolo[3,4-*b*]quinolin-3(1*H*)-one	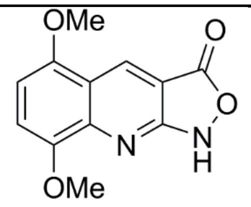
**24**	1,7-Dimethylisoxazolo[3,4-*b*]quinolin-3(1*H*)-one	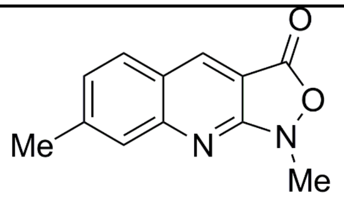
**25**	5,8-Dimethoxy-1-methylisoxazolo[3,4-*b*]quinolin-3(1*H*)-one	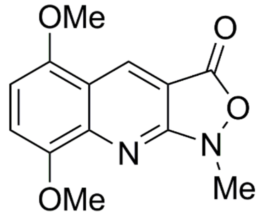
**26**	1,6-Dimethylisoxazolo[3,4-*b*]quinolin-3(1*H*)-one	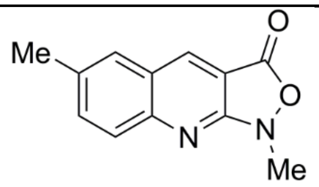

Table S2. The retention data of the studied pyrido- and quinolino-isoxazolones obtained by IAM-HPLC

No.	T_{R1}	T_{R2}	T_{R3}	*SD*
1	2.379	2.299	2.303	0.045
2	2.350	2.395	2.407	0.030
3	3.137	3.165	3.156	0.014
4	2.958	2.976	2.976	0.010
5	2.302	2.293	2.301	0.005
6	3.734	3.747	3.747	0.008
7	2.498	2.518	2.521	0.013
8	2.971	2.978	2.985	0.007
9	3.479	3.491	3.481	0.006
10	2.623	2.651	2.651	0.016
11	3.734	3.748	3.740	0.007
12	2.293	2.283	2.282	0.006
13	3.753	3.764	3.767	0.007
14	*nd*	*nd*	*nd*	-
15	3.387	3.404	3.402	0.009
16	2.919	2.936	2.935	0.010
17	2.009	2.054	2.001	0.029
18	3.348	3.368	3.358	0.010
19	3.360	3.363	3.363	0.002
20	3.262	3.268	3.269	0.004
21	3.702	3.706	3.703	0.002
22	3.726	3.731	3.727	0.003
23	2.546	2.546	2.551	0.003
24	3.464	3.470	3.476	0.006
25	3.342	3.350	3,344	0,004
26	3.507	3.512	3,506	0,003

nd – not detected

Table S3. The IAM-HPLC retention data of model substances used for determination of the CHI_{IAM} values, along with information regarding suppliers

	T_{R1}	T_{R2}	T_{R3}	*SD*	supplier
Octanophenone	4.760	4.761	4.762	0.001	Alfa Aesar[1]
Heptanophenone	4.528	4.529	4.530	0.001	Acros Organic[3]
Hexanophenone	4.266	4.268	4.269	0.002	Acros Organic[3]
Valerophenone	3.956	3.958	3.959	0.002	Acros Organic[3]
Butyrophenone	3.585	3.588	3.590	0.003	Alfa Aesar[1]
Propiohenone	3.149	3.152	3.156	0.004	Acros Organic[3]
Acetophenone	2.578	2.583	2.587	0.005	Sigma-Aldrich[2]
Acetanilidine	2.284	2.288	2.295	0.006	Alfa Aesar[1]
Paracetamol	1.823	1.825	1.832	0.005	Sigma-Aldrich[2]

[1]-Alfa Aesar (Haverhill, MA, USA)

[2]-Sigma-Aldrich (Steinheim, Germany)

[3]-Acros Organic (Massachusetts, United States).

© 2020 by the authors. Licensee MDPI, Basel, Switzerland. This article is an open access article distributed under the terms and conditions of the Creative Commons Attribution (CC BY) license (http://creativecommons.org/licenses/by/4.0/).
